# Management of Recurrent Glioblastomas: What Can We Learn from the French Glioblastoma Biobank?

**DOI:** 10.3390/cancers14225510

**Published:** 2022-11-09

**Authors:** Anne Clavreul, Lila Autier, Jean-Michel Lemée, Paule Augereau, Gwénaëlle Soulard, Luc Bauchet, Dominique Figarella-Branger, Philippe Menei, FGB Network

**Affiliations:** 1 Département de Neurochirurgie, CHU, 49933 Angers, France; 2 Université d’Angers, Inserm UMR 1307, CNRS UMR 6075, Nantes Université, CRCINA, F-49000 Angers, France; 3 Département de Neurologie, CHU, 49933 Angers, France; 4 Département d’Oncologie Médicale, Institut de Cancérologie de l’Ouest, Site Paul Papin, 49055 Angers, France; 5 Département de Neurochirurgie, Hôpital Gui de Chauliac, CHU Montpellier, Université de Montpellier, 34295 Montpellier, France; 6 Institut de Génomique Fonctionnelle, CNRS, INSERM, 34295 Montpellier, France; 7 APHM, CHU Timone, Service d’Anatomie Pathologique et de Neuropathologie, 13385 Marseille, France; 8 Aix-Marseille University, CNRS, INP, Inst. Neurophysiopathol, 13005 Marseille, France

**Keywords:** IDH wild-type, prognosis, recurrent glioblastoma, reoperation, survival, systemic treatment

## Abstract

**Simple Summary:**

There is no broad consensus concerning the management of recurrent glioblastoma (rGB). Within the French GB biobank (FGB), systemic treatment is the principal second-line treatment. None of the systemic treatment regimens was unequivocally better than the others for rGB patients. An analysis of survival outcomes based on time to first recurrence (TFR) showed that survival was best in patients with a long TFR, but that these patients constituted only a small proportion of rGB patients (13.0%). This better survival appears to be more strongly associated with response to first-line treatment than with response to second-line treatment, indicating that recurring tumors are more aggressive and/or resistant than the initial tumors in these patients. In the face of high rates of treatment failure for GB, the establishment of well-designed large cohorts of primary and rGB samples, with the help of biobanks, such as the FGB, is urgently required for the performance of solid comparative biological analyses to drive the development of new therapies for GB.

**Abstract:**

Safe maximal resection followed by radiotherapy plus concomitant and adjuvant temozolomide (TMZ) is universally accepted as the first-line treatment for glioblastoma (GB), but no standard of care has yet been defined for managing recurrent GB (rGB). We used the French GB biobank (FGB) to evaluate the second-line options currently used, with a view to defining the optimal approach and future directions in GB research. We retrospectively analyzed data for 338 patients with de novo isocitrate dehydrogenase (IDH)-wildtype GB recurring after TMZ chemoradiotherapy. Cox proportional hazards models and Kaplan–Meier analyses were used to investigate survival outcomes. Median overall survival after first surgery (OS1) was 19.8 months (95% CI: 18.5–22.0) and median OS after first progression (OS2) was 9.9 months (95% CI: 8.8–10.8). Two second-line options were noted for rGB patients in the FGB: supportive care and treatments, with systemic treatment being the treatment most frequently used. The supportive care option was independently associated with a shorter OS2 (*p* < 0.001). None of the systemic treatment regimens was unequivocally better than the others for rGB patients. An analysis of survival outcomes based on time to first recurrence (TFR) after chemoradiotherapy indicated that survival was best for patients with a long TFR (≥18 months; median OS1: 44.3 months (95% CI: 41.7–56.4) and median OS2: 13.0 months (95% CI: 11.2–17.7), but that such patients constituted only a small proportion of the total patient population (13.0%). This better survival appeared to be more strongly associated with response to first-line treatment than with response to second-line treatment, indicating that the recurring tumors were more aggressive and/or resistant than the initial tumors in these patients. In the face of high rates of treatment failure for GB, the establishment of well-designed large cohorts of primary and rGB samples, with the help of biobanks, such as the FGB, taking into account the TFR and survival outcomes of GB patients, is urgently required for solid comparative biological analyses to drive the discovery of novel prognostic and/or therapeutic clinical markers for GB.

## 1. Introduction

Glioblastoma (GB) is the most common primary intracranial malignancy, accounting for 30% of all central nervous system tumors, with an incidence of 3.22 per 100,000 individuals [1]. The standard of care for patients with primary isocitrate dehydrogenase (IDH)-wildtype GB is maximal safe surgical resection, when feasible, followed by concomitant chemoradiotherapy and six cycles of temozolomide (TMZ) treatment (EORTC 26981-NCIC CE.3) [2,3]. The prognosis remains poor, even with treatment, with a median survival of about 15 months and a 5-year survival rate of 4% [4,5]. Tumor relapse almost invariably occurs at or close to the initial site of disease [6,7,8].

There is universal agreement about the first-line treatment for primary GB, but the best way to manage recurrent GB (rGB) is less clear, given that none of the treatments used for recurrences has ever been shown to be more beneficial than the others [3,9,10,11,12,13,14,15]. The management of rGB is based on expert guidelines, such as those of the European Society for Medical Oncology (ESMO) [16], the European Association of Neuro-Oncology (EANO) [3], the National Comprehensive Cancer Network (NCCN, https://www.nccn.org (accessed on 1 February 2022)) and “Association des Neuro-Oncologues d’Expression Française” (ANOCEF, https://www.anocef.org (accessed on 1 January 2018)). Treatment decisions often require multi-disciplinary discussions on a case-by-case basis, to determine the optimal second-line options for improving survival and health-related quality of life (HRQoL).

The aim of this retrospective study was to use French GB biobank (FGB) data to evaluate, retrospectively, the second-line options currently used, with a view to defining the optimal approach and future directions in GB research. The FGB is an academic biobank developed in 2012, following a call for tenders from the “Institut National du Cancer” (INCa) [17]. This biobank holds biological materials and clinical data for adult patients with GB, and it is managed with the support of neurosurgeons, neuropathologists, neuro-oncologists and biologists from 25 centers throughout France. Clinical data from about 1400 GB patients, including epidemiological, imaging, tumor characteristics and follow-up data, have been included in the FGB to date, together with a collection of biological samples, including frozen and formalin-fixed paraffin-embedded tumor tissues and blood samples. We selected the patients with de novo IDH-wildtype GB recurring after surgical resection and TMZ chemoradiotherapy for this retrospective analysis.

## 2. Patients and Methods

### 2.1. Patients

This retrospective study focused on patients included in the FGB biobank after the diagnosis of IDH-wildtype GB between January 2012 and December 2020. The following inclusion criteria were used: (1) patient aged ≥18 years, (2) newly diagnosed unilateral supratentorial GB, (3) GB with negative immunohistochemical staining for IDH1-R132H, (4) tumor resected, (5) no intraoperative chemotherapy, (6) first-line treatment according to the Stupp protocol (this protocol consists of focal irradiation fractionated into daily doses of 2 Gy administered five days/week for six weeks, for a total of 60 Gy, plus concomitant daily TMZ (75 mg/m^2^/day, 7 days/week from the first to the last day of radiotherapy), followed by six cycles of adjuvant TMZ (150–200 mg/m^2^/day for 5 days during each 28-day cycle)) and (7) clinical or radiological evidence of progression after concurrent chemoradiotherapy, according to the RANO criteria [18]. Patients with a history of tumors preceding the tumor for which they were included in the database were excluded. Patients who underwent biopsies were also excluded, because such patients constitute only a small percentage of the patients included in the FGB due to insufficient amounts of tumor tissue for storage. Based on these criteria, we included a total of 338 patients.

### 2.2. Eligibility and Informed Consent

The FGB network was declared to the French Ministry of Health and Research (declaration number: DC-2011-1467, cession authorization number: AC-2017-2993). The protocols and regulations of the FGB network were approved by the CPP OUEST II ethics committee (CB 2012/02, date of approval: 20 December 2011) and the CNIL (“Commission Nationale de l’Informatique et des Libertés”, the French national data protection authority, no. 1476342, date of approval: 10 October 2011). All adult patients from this retrospective analysis signed an informed consent form for the inclusion of their data and samples in the biobank.

### 2.3. Data Collection

Baseline characteristics, such as age, sex, preoperative Karnofsky performance score (KPS), tumor laterality, tumor extent, extent of resection (EOR), O(6)-methylguanine methyltransferase (MGMT) methylation status, Stupp protocol regimen, recurrence location and follow-up, were collected from eCRFs built with Ennov Clinical software (Ennov, Paris, France). This software is ISO9001:2015-certified for all products and activities and meets the recommendations of the FDA 21CRF Part11 and the EMA for the IT security of clinical data. The methylation status of the MGMT promoter was assessed, according to local standards, by methylation-specific PCR or pyrosequencing. EOR was recorded by the surgeon performing the operation or was determined from a postoperative MRI scan performed within 48 h of surgery by a neuroradiologist. EOR was classified as gross total resection (GTR; 100%), subtotal resection (STR; ≥90%) or partial resection (PR; <90%). EOR1 was the extent of the initial tumor resection and EOR2, the extent of the second tumor resection. The recurrence was considered local if it occurred at the same site as the initial tumor and distant if it had spread to another site. Time to first recurrence (TFR) was defined as the time between the date on which chemoradiotherapy ended and the date of first progression. TFR was classified as follows: short TFR (≤6 months), intermediate TFR (7 to 17 months) and long TFR (≥18 months). Progression-free survival (PFS1) was measured from the date of initial surgery to the date of first progression. PFS2 was defined as the time between the first and second progression. Overall survival (OS1) was calculated from the date of initial surgery to the date of last follow-up or death. OS2 was measured from the date of first progression to the date of last follow-up or death. Patients alive at last follow-up were censored.

### 2.4. Statistical Analysis

Differences between groups were assessed with Chi-squared tests, Fisher’s exact tests, ANOVA or Kruskal–Wallis tests, as appropriate. *p*-values were adjusted by the Benjamini–Hochberg (BH) method for multiple testing. Spearman’s rank correlation analyses were performed to evaluate the relationship between two variables. Univariate Cox regression analysis was performed with the covariates of all patients to screen for factors associated with OS1 and OS2. Variables with raw *p*-values < 0.25 in univariate analysis were included in multivariate Cox regression analysis, unless they were correlated with each other. The global statistical significance of the Cox model was checked in three alternative tests (likelihood ratio, Wald and log-rank tests). The Cox model was also tested by two types of diagnostics: Schoenfeld residuals, to check the assumption of proportional hazards, and the determination of dfbeta values to investigate influential outliers. Survival curves were plotted according to the Kaplan–Meier method and were compared in log-rank tests. Statistical analyses were performed with R software (version 4.1.0; https://cran.r-project.org (accessed on 18 May 2021)). Values of *p* < 0.05 were considered statistically significant.

## 3. Results

### 3.1. General Characteristics of rGB Patients and Second-Line Treatment Options

The baseline characteristics of the 338 selected IDH-wildtype GB patients are shown in Table 1. Median age at diagnosis was 61 years, and 223 patients (66.0%) were male. In total, 223 patients (66.0%) had a KPS score > 70% before surgery. The GB was in the left hemisphere in 151 patients (44.7%) and the right hemisphere in 187 patients (55.3%). GB was unilobar in 211 patients (62.4%) and multilobar in 127 patients (37.6%). EOR1 was complete in 172 patients (50.9%). MGMT promoter status was available for 168 patients (49.7%), and 72 GB (42.9%) displayed MGMT methylation. All patients received concurrent chemoradiotherapy according to the Stupp protocol after initial surgery. However, 206 patients (60.9%) underwent fewer than six cycles of adjuvant TMZ and 132 patients (39.1%) underwent six or more cycles. Relapses were recorded in all patients, and the recurrence was local in 299 patients (88.5%). TFR was short (≤6 months) in 210 patients (62.0%), intermediate (7 to 17 months) in 84 patients (25.0%) and long (≥18 months) in 44 patients (13.0%). In pairwise comparisons with BH correction for multiple testing, the intermediate TFR group was found to include more patients with GB in the right hemisphere than the short TFR and long TFR groups (post hoc *p* = 0.048 and post hoc *p* = 0.018, respectively). The short TFR group contained more patients with multilobar GB and incomplete first resection than the long TFR group (post hoc *p* = 0.003 and post hoc *p* = 0.049, respectively). The short TFR group contained more patients with short courses of adjuvant TMZ treatment than the intermediate TFR and long TFR groups (post hoc *p* < 0.001, for the two). GB with methylated MGMT promoters were more frequent in the long TFR group than in the short TFR and intermediate TFR groups (post hoc *p* < 0.001 and post hoc *p* = 0.018, respectively).

**Figure 1:**
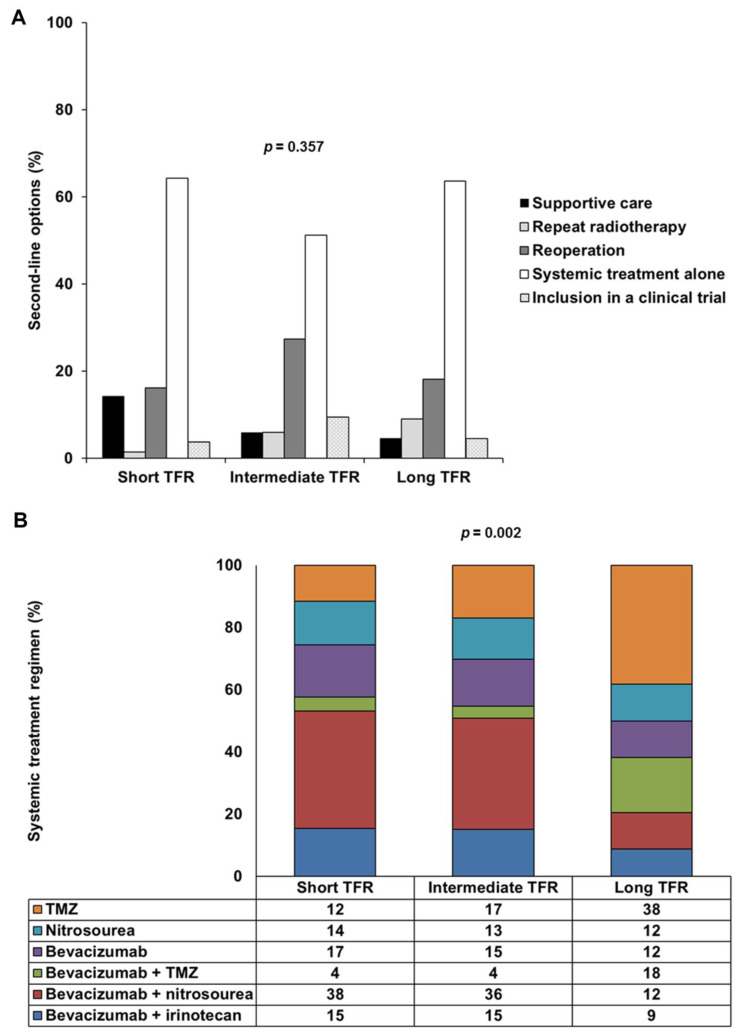
Second-line options and systemic treatment regimens used, by TFR: short TFR (≤6 months), intermediate TFR (7 to 17 months) and long TFR (≥18 months). (**A**) Frequency of five types of second-line options (supportive care, repeat radiotherapy, reoperation, systemic treatment alone or inclusion in a clinical trial) in the short TFR, intermediate TFR and long TFR groups. (**B**) Frequency of the six main types of systemic treatment regimen used (TMZ, nitrosourea, bevacizumab, bevacizumab plus TMZ, bevacizumab plus nitrosourea and bevacizumab plus irinotecan) in the short TFR, intermediate TFR and long TFR groups. Abbreviations: TFR, time to first recurrence; TMZ, temozolomide.

As second-line treatment options, 37 patients (10.9%) had supportive care and 301 patients (89.1%) received treatment after the first progression. Of the 301 patients treated, 12 (3.6%) underwent repeat radiotherapy, 65 patients (19.2%) underwent reoperation, including 26 (7.7%) who received intratumoral treatment consisting of carmustine-releasing wafers (Gliadel^®^), 206 patients (60.9%) underwent systemic treatment alone and 18 patients (5.3%) were included in clinical trials. There were six principal systemic treatment regimens: TMZ rechallenge, nitrosourea monotherapy and bevacizumab alone or combined with TMZ, nitrosourea or irinotecan. Lomustine was the principal nitrosourea, used in 81.7% of cases. There was no significant difference in second-line options between the three TFR-based groups (*p* = 0.357), but systemic treatment regimens differed significantly between the long TFR group and the short TFR and intermediate TFR groups (post hoc *p* = 0.002 and post hoc *p* = 0.001, respectively) (Table 1 and Figure 1). TMZ or bevacizumab plus TMZ was administered more frequently than bevacizumab plus nitrosourea in the long TFR group.

### 3.2. Survival Outcomes of rGB Patients

The 338 patients had a median PFS1 of 7.8 months (95% CI: 6.8–8.6) and a median OS1 of 19.8 months (95% CI: 18.5–22.0) (Table 1). After second-line treatment, the median PFS2 was 5.5 months (95% CI: 4.8–6.0) and the median OS2 was 9.9 months (95% CI: 8.8–10.8).

**Figure 2:**
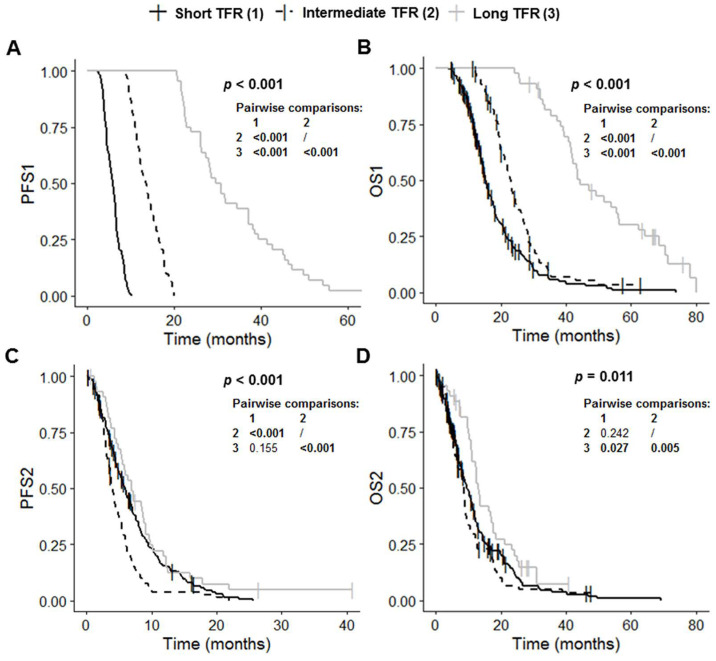
Kaplan–Meier curves for survival stratified for TFR: short TFR (≤6 months, *n* = 210), intermediate TFR (7 to 17 months, *n* = 84) and long TFR (≥18 months, *n* = 44). (**A**) PFS1, (**B**) OS1, (**C**) PFS2, and (**D**) OS2. Abbreviations: OS1, overall survival after first surgery; OS2, overall survival after first progression; PFS1, progression-free survival after first surgery; PFS2, progression-free survival after first progression; TFR, time to first recurrence.

The short TFR, intermediate TFR and long TFR groups had significantly different PFS1 (*p* < 0.001), OS1 (*p* < 0.001), PFS2 (*p* < 0.001) and OS2 (*p* = 0.011) values (Table 1 and Figure 2). Post-hoc tests with BH correction showed that PFS1 in the long TFR group (30.2 months (95% CI: 27.4–37.1)) was significantly longer than that in the short TFR (5.9 months (95% CI: 5.4–6.3); post hoc *p* < 0.001) and intermediate TFR groups (13.6 months (95% CI: 12.2–14.9); post hoc *p* < 0.001) (Figure 2A). The PFS1 of the intermediate TFR group was also significantly longer than that in the short TFR group (post hoc *p* < 0.001) (Figure 2A). Similarly, OS1 in the long TFR group (44.3 months (95% CI: 41.7–56.4) was significantly longer than that in the short TFR (15.2 months (95% CI: 14.4–17.1); post hoc *p* < 0.001) and intermediate TFR groups (22.9 months (95% CI: 21.2–25.1); post hoc *p* < 0.001) (Figure 2B). OS1 in the intermediate TFR group was also significantly longer than that in the short TFR group (post hoc *p* < 0.001) (Figure 2B). PFS2 in the intermediate TFR group (4.0 months (95% CI: 3.5–4.9) was significantly shorter than that in the short TFR (5.9 months (95% CI: 5.1–6.9); post hoc *p* < 0.001) and long TFR groups (7.0 months (95% CI: 5.5–9.1); post hoc *p* < 0.001) (Figure 2C). PFS2 did not differ significantly between the short TFR and long TFR groups (post hoc *p* = 0.155). OS2 in the long TFR group (13.0 months (95% CI: 11.2–17.7)) was significantly longer than that in the short TFR (9.5 months (95% CI: 8.3–11.2); post hoc *p* = 0.027) and intermediate TFR groups (8.5 months (95% CI: 6.7–9.3); post hoc *p* = 0.005) (Figure 2C). OS2 did not differ significantly between the short TFR and intermediate TFR groups (post hoc *p* = 0.242).

Eight variables were associated with a shorter OS1 in univariate analysis: older age (*p* = 0.001), multilobar tumor (*p* = 0.008), unmethylated MGMT (*p* < 0.001), short period of TMZ consolidation treatment (*p* < 0.001), short PFS1 (*p* < 0.001), short or intermediate TFR (vs. long TFR) (*p* < 0.001), short PFS2 (*p* < 0.001) and the supportive care option (*p* < 0.001) (Table S1). Five variables were associated with a shorter OS2 in univariate analysis: older age (*p* = 0.010), unmethylated MGMT (*p* < 0.001), short or intermediate TFR (vs. long TFR) (*p* = 0.018 and *p* = 0.003, respectively), short PFS2 (*p* < 0.001) and the supportive care option (*p* < 0.001) (Table S1).

TFR was significantly positively correlated with PFS1, the number of cycles of adjuvant TMZ and MGMT methylation status (Table S2). No correlation was found between TFR and PFS2 (Table S2). The Cox model for OS1 was stratified for TFR and PFS2, whereas that for OS2 was stratified for PFS2, to satisfy the assumption of proportional hazards. We identified two variables as independently associated with shorter OS1: older age (*p* = 0.007) and the supportive care option (*p* < 0.001) (Table 2). One variable was independently associated with a shorter OS2: the supportive care option (*p* < 0.001) (Table 2). The OS2 of rGB patients receiving supportive care was 2.9 months (95% CI: 2.2–4.1), whereas that of patients receiving some kind of treatment was 10.6 months (95% CI: 9.5–11.9) (*p* < 0.001) (Table 3). Older age, being male and a short TFR (vs. long TFR) tended to be associated with a poorer OS2 (*p* = 0.094, *p* = 0.097 and *p* = 0.089, respectively).

### 3.3. Analysis of the Efficacy of Second-Line Treatments

Given the diversity of second-line treatments, two main analyses were conducted: one to compare systemic treatment with and without reoperation and the other to compare the efficacy of repeat surgery with and without the intratumoral treatment with Gliadel^®^. It was not possible to stratify these two analyses for TFR due to the small number of patients with intermediate or long TFR.

#### 3.3.1. Comparison of Systemic Treatment with and without Reoperation (*n* = 227)

We considered the six systemic treatments most frequently prescribed: TMZ rechallenge, nitrosourea monotherapy and bevacizumab alone or combined with TMZ, nitrosourea or irinotecan. Patients who underwent repeat radiotherapy or reoperation with intratumoral Gliadel^®^ treatment were excluded from the analysis. The characteristics of the patients retained for the analysis are shown in Table S3. In total, 227 patients received systemic treatment: 32 (14.1%) were treated with TMZ, 32 (14.1%) with nitrosourea, 36 (15.9%) with bevacizumab, 14 (6.2%) with bevacizumab plus TMZ, 80 (35.2%) with bevacizumab plus nitrosourea and 33 (14.5%) with bevacizumab plus irinotecan. Pairwise comparisons with BH correction for multiple testing showed that the proportion of distant rGB was significantly higher in the bevacizumab plus TMZ group than in the TMZ, nitrosourea, bevacizumab plus nitrosourea and bevacizumab plus irinotecan groups (post hoc *p* = 0.027 for all comparisons). Reoperation was more frequent in the nitrosourea group than in the bevacizumab and bevacizumab plus nitrosourea groups (post hoc *p* = 0.013 and post hoc *p* = 0.022, respectively). Reoperation rates were also higher in the TMZ group than in the bevacizumab group (post hoc *p* = 0.023). The TFR of the bevacizumab plus nitrosourea group was significantly different from those of the TMZ (post hoc *p* = 0.004) and bevacizumab plus TMZ (post hoc *p* = 0.046) groups, the bevacizumab plus nitrosourea group having fewer patients with a long TFR.

**Figure 3:**
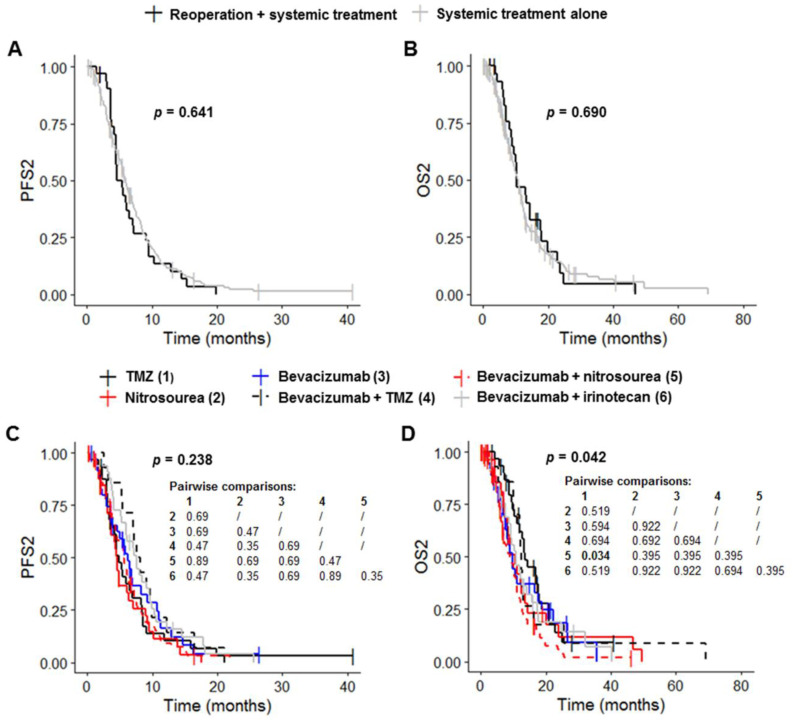
Survival outcomes for patients on systemic treatment. (**A**,**B**) Kaplan–Meier curves for survival stratified for systemic treatment with reoperation (*n* = 32) and systemic treatment without reoperation (*n* = 195) ((**A**) PFS2; (**B**) OS2). (**C**,**D**) Kaplan–Meier curves for survival stratified for the six most frequent systemic treatment regimens: TMZ rechallenge (*n* = 32), nitrosourea monotherapy (*n* = 32), bevacizumab alone (*n* = 36) or combined with TMZ (*n* = 14), nitrosourea (*n* = 80) and irinotecan (*n* = 33) ((**C**) PFS2; (**D**) OS2). Abbreviations: OS2, overall survival after first progression; PFS2, progression-free survival after first progression; TMZ, temozolomide.

Patients receiving systemic treatment without reoperation had a median PFS1 of 6.7 months (95% CI: 6.5–7.9), a median OS1 of 20.5 months (95% CI: 18.5–23.0), a median PFS2 of 6.0 months (95% CI: 5.4–6.9) and a median OS2 of 10.8 months (95% CI: 9.4–12.0) (Table 3, Figure 3A,B and Figure S1A,B). Reoperation before systemic treatment had no significant effect on PFS1, OS1, PFS2 and OS2 (*p* = 0.841, *p* = 0.945, *p* = 0.641 and *p* = 0.690, respectively) (Table 3, Figure 3A,B and Figure S1A,B). PFS1 and OS1 differed significantly between the six systemic treatment groups (*p* = 0.004 and *p* < 0.001, respectively) (Table 3 and Figure S1C). Post hoc tests with BH correction revealed that the PFS1 of the Bevacizumab plus nitrosourea group (6.7 months (95% CI: 6.5–7.4) was significantly shorter than that of the TMZ group (13.4 months (95% CI: 6.2–22.6); post hoc *p* = 0.009) (Figure S1C). OS1 in the bevacizumab plus nitrosourea group (18.2 months (95% CI: 15.2–20.8)) was also significantly shorter than that in the TMZ (30.3 months (95% CI: 23.5–44.3); post hoc *p* < 0.001) and bevacizumab plus TMZ groups (34.6 months (95% CI: 17.3–NA); post hoc *p* = 0.016) (Table 3 and Figure S1D). The use of treatment regimens other than TMZ or bevacizumab plus TMZ had no significant effect on OS1 change. Median OS1 was 22.2 months (95% CI: 16.2–33.5) for the nitrosourea group, 21.4 months (95% CI: 17.6–35.4) for the bevacizumab group and 18.5 months (95% CI: 16.2–28.0) for the bevacizumab plus irinotecan group (Table 3). PFS2 did not differ significantly between the six groups (*p* = 0.238), although there was a trend towards longer PFS2 in the groups receiving bevacizumab (Figure 3C and Table 3). Median PFS2 was 4.8 months (95% CI: 3.7–8.3) in the TMZ group, 4.6 months (95% CI: 3.6–7.1) in the nitrosourea group, 6.2 months (95% CI: 4.6–10.8) in the bevacizumab group, 7.9 months (95% CI: 7.0–16.5) in the bevacizumab plus TMZ group, 5.6 months (95% CI: 4.6–6.9) in the bevacizumab plus nitrosourea group and 7.5 months (95% CI: 5.9–9.6) in the bevacizumab plus irinotecan group. OS2 differed significantly between the six groups (*p* = 0.042) (Figure 3D and Table 3). Post hoc tests with BH correction showed that OS2 was significantly shorter in the bevacizumab plus nitrosourea group (8.8 months (95% CI: 6.9–11.1)) than in the TMZ group (13.5 months (95% CI: 12.0–20.8); post hoc *p* = 0.034). OS2 did not differ significantly between the TMZ and other treatment groups. Median OS2 was 10.6 months (95% CI: 8.2–14.2) in the nitrosourea group, 9.9 months (95% CI: 7.5–18.7) in the bevacizumab group, 12.4 months (95% CI: 10.1–NA) in the bevacizumab plus TMZ group and 10.8 months (95% CI: 9.1–17.4) in the bevacizumab plus irinotecan group.

#### 3.3.2. Reoperation with and without Gliadel^®^ (*n* = 58)

Patients who underwent reoperation and received systemic treatment (reoperation plus systemic treatment, *n* = 32) from the previous analysis were compared with patients undergoing reoperation with intratumoral Gliadel^®^ treatment (reoperation plus Gliadel^®^, *n* = 26). The characteristics of the selected patients are shown in Table S4. Of the 26 patients treated with Gliadel^®^, 9 patients (34.6%) also received systemic treatment. 

**Figure 4:**
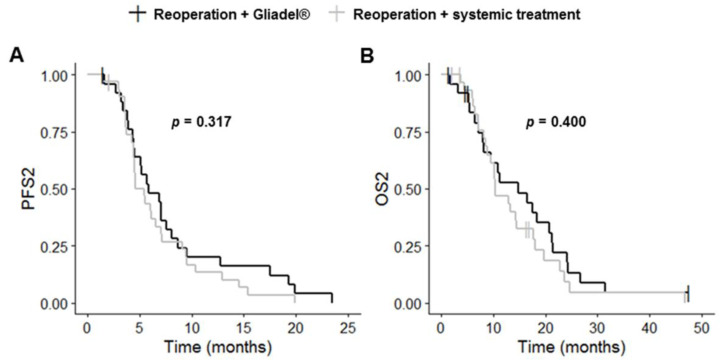
Kaplan–Meier curves for survival stratified for reoperation with Gliadel^®^ (*n* = 26) and reoperation with systemic treatment (*n* = 32) ((**A**) PFS2; (**B**) OS2). Abbreviations: OS2, overall survival after first progression; PFS2, progression-free survival after first progression.

Complete resection of the primary and recurrent tumors was more frequent in the reoperation plus Gliadel^®^ group than in the reoperation plus systemic treatment group (*p* = 0.003 and *p* < 0.001, respectively). PFS1, OS1, PFS2 and OS2 did not differ significantly between these two groups (*p* = 0.562, *p* = 0.423, *p* = 0.303 and *p* = 0.436, respectively) (Table 3, Figure 4 and Figure S2). The reoperation plus Gliadel^®^ group had a median PFS1 of 8.6 months (95% CI: 5.9–12.2), a median OS1 of 24.4 months (95% CI: 18.3–33.0), a median PFS2 of 5.9 months (95% CI: 4.5–8.7) and a median OS2 of 14.8 months (95% CI: 8.1–21.4).

## 4. Discussion

There is no widely accepted approach for managing rGB after TMZ chemoradiotherapy. The second-line option is, therefore, based on the preferences of the treating center, the individual characteristics of the patient and tumor aggressiveness, which can be assessed by determining TFR after chemoradiotherapy [3,19]. In this study, a short TFR (≤6 months) was observed in 210 patients (62.0%), whereas intermediate (7 to 17 months) and long (≥18 months) TFRs were observed in only 84 (25.0%) and 44 patients (13.0%), respectively. As expected, TFR was strongly and significantly correlated with PFS1, with a median PFS1 of 5.9 months for short TFR, 13.6 months for intermediate TFR and 30.2 months for long TFR. TFR was also positively correlated with the number of cycles of adjuvant TMZ and MGMT methylation status. The standard guidelines recommend six months of adjuvant TMZ, but this treatment can be extended to more than six cycles, as shown by the FGB data. The impact of prolonging maintenance TMZ therapy beyond six cycles remains a matter of debate [20,21,22]. This retrospective study was not designed to determine the survival benefits of prolonged adjuvant TMZ over the standard six-cycle TMZ regimen in patients with GB. Furthermore, the absence of MGMT methylation status for more than 50% of the patients would greatly limit the strength of any conclusions drawn. Nevertheless, we observed that 17 (40.5%) of the GB patients with a long TFR receiving six or more cycles of adjuvant TMZ (*n* = 42) were on the standard six-cycle regimen and that 25 patients (59.5%) received adjuvant TMZ treatment for a prolonged period, indicating that a reasonable proportion of patients on standard maintenance therapy may have a long TFR.

The 338 GB patients included in this retrospective study had a median OS1 after first surgery of 19.8 months and a median OS2 after first progression of 9.9 months; these values are typical for GB patients in the Stupp era [23,24,25]. The patients with a long TFR had a significantly longer OS1 and OS2 than those with a short or intermediate TFR (median OS1: 44.3 vs. 15.2 and 22.9 months, respectively; median OS2: 13.0 vs. 9.5 and 8.5 months, respectively). Patients with a long TFR also had a significantly longer PFS2 than patients with an intermediate TFR (median PFS2: 7.0 vs. 4.0 months) but, surprisingly, no significant difference in PFS2 was observed between patients with a long TFR and those with a short TFR (median PFS2: 7.0 vs. 5.9 months). This finding may be explained by the higher risks of pseudoprogression in patients with a short TFR. OS2 was about 4 to 5 months longer in patients with a long TFR than in those with a short or intermediate TFR, but this is a small difference relative to that for PFS1 (about 16–24 months longer for patients with a long TFR). Similar post-progression survival was previously reported between GB patients with and without MGMT promoter methylation [9]. These results suggest that the better survival of patients with a long TFR or with GB displaying MGMT-promoter methylation is mostly due to their response to first-line treatment rather than their response to second-line treatment. They also indicate that the rGB of these patients were more aggressive and/or resistant than their initial tumors.

A multivariate Cox regression analysis of factors associated with OS2 showed that the supportive care option after first progression was a significant independent predictor of shorter OS2. Being older, male and having a short TFR also tended to be independent predictors of poor OS2. The survival disadvantage of these factors after progression has already been highlighted in other studies [19,24,26,27]. However, by contrast to previous studies, KPS at diagnosis and EOR1 were not found to be independent factors associated with OS2 in this cohort [26,28,29,30]. The lack of accurate KPS score determinations and of quantitative MRI assessments of volume for EOR evaluation may account for this discrepancy. We were unable to analyze KPS at progression and MGMT methylation status, which were found to be predictors of OS2 in previous studies [26,31] in multivariate Cox regression analysis due to the large amounts of missing data.

The decision to give supportive care rather than treatment after a first progression is generally based on rapid progression of the GB associated with comorbid conditions and/or a worsening of neurological state. Older age, contraindications for alternative options or the patient’s refusal to prolong therapy may also underlie the decision to implement supportive care. Systemic treatment alone was the most common second-line treatment recorded in the FGB, this treatment being administered to 206 patients (60.9%). Repeat radiotherapy was performed in only 12 patients (3.6%). This treatment option may continue to be limited because unanswered questions remain about the efficacy and toxicity of a second course of radiation, even with improvements in the techniques used, such as stereotactic radiosurgery and stereotactic radiotherapy [32]. Reoperation was indicated in 69 patients (20.4%), consistent with published findings suggesting that 20–30% of patients undergo reoperation [3,33]. The remaining 18 patients (5.3%) were included in clinical trials. A significant difference in systemic treatment regimens was observed between the three TFR groups. TMZ rechallenge and bevacizumab plus TMZ were administered more frequently to patients with a long TFR, whereas bevacizumab plus nitrosourea was prescribed more frequently for patients with short and intermediate TFR. This is not surprising given that the patients with a long TFR were those who responded most successfully to first-line Stupp treatment.

Given the small number of patients with intermediate or long TFR, all 338 GB patients selected from the FGB database were included in the analysis of second-line treatment efficacy. This analysis showed that reoperation before the use of systemic treatment did not increase survival. Median OS2 was 10.4 for the reoperated and 10.8 months for the non-reoperated groups. Similarly, there was no significant difference in survival between rGB patients undergoing reoperation with intratumoral Gliadel^®^ treatment and rGB patients undergoing reoperation and receiving systemic treatment (median OS2: 14.8 vs. 10.4 months). The benefits of repeat resection after the first recurrence remain unclear from published data [34]. Some studies have suggested that there is a survival benefit after reoperation, whereas others found no such benefit [35,36,37]. A lack of randomization, selection bias in patient inclusion and exclusion, study timing (pre- or post-Stupp protocol era), different treatments after further resection and differences in the definition of OS may account for the discrepancies between studies. Additional well-designed studies are now required to determine the real benefit of reoperation with or without intratumoral treatment. It was not possible to analyze the survival benefit of repeat radiotherapy in our study due to the small number of patients receiving this treatment. Repeat radiotherapy is currently considered safe for rGB management, but the heterogeneity of studies in terms of patient characteristics and radiotherapy regimen makes it difficult to draw any firm conclusions about the efficacy of this treatment option for rGB patients [32,38,39]. For systemic treatments, OS2 did not differ significantly between patients receiving bevacizumab plus nitrosourea and those treated with nitrosourea alone (median OS2: 8.8 vs. 10.6 months) or bevacizumab alone (median OS2: 8.8 vs. 9.9 months). Two previous clinical trials—a phase III trial with 437 rGB patients [40] and a phase II TAMIGA trial with 123 rGB patients [41]—showed that lomustine plus bevacizumab provided no significant improvement in OS after recurrence over lomustine monotherapy. Similarly, Weathers et al. [42] showed, in a phase II trial, that the low-dose bevacizumab plus lomustine combination was not superior to standard-dose bevacizumab in 69 patients with rGB. We also observed that patients who received bevacizumab plus irinotecan and, thus, who received bevacizumab alone had similar outcomes (median OS2: 10.8 vs. 9.9 months). These data are consistent with the findings of Friedman et al. [43], who evaluated the efficacy of bevacizumab, alone and in combination with irinotecan, in patients with rGB in a phase II, multicenter, open-label, noncomparative trial (*n* = 167). Median OS from randomization was 9.2 months and 8.7 months, respectively. Furthermore, similar to Jakobsen et al. [44], we found no significant difference in median OS2 between bevacizumab plus nitrosourea and bevacizumab plus irinotecan (median OS2: 8.8 vs. 10.8 months). The only significant difference in OS2 observed in this study was that between patients receiving bevacizumab plus nitrosourea and those prescribed TMZ rechallenge or bevacizumab plus TMZ (median OS2: 8.8 vs. 13.5 and 12.4 months, respectively). Other treatment regimens were no more effective than TMZ rechallenge or bevacizumab plus TMZ. The longer OS2 observed with TMZ rechallenge and with bevacizumab plus TMZ than with bevacizumab plus nitrosourea may result from a bias, because the TMZ rechallenge and bevacizumab plus TMZ groups contained more patients with a long TFR than the bevacizumab plus nitrosourea group. One study retrospectively compared data from rGB patients completing standard TMZ treatment concurrently and adjuvant to radiotherapy and undergoing TMZ rechallenge or nitrosourea treatment after progression [45]. The authors showed that, for patients with rGB after a treatment-free interval of at least five months, median OS2 and PFS2 were longer for the TMZ rechallenge group than for the nitrosourea group, regardless of the MGMT methylation status of the tumor. Median OS2 was 17.7 months for the TMZ group and 11.6 months for the nitrosourea group, and median PFS2 was 8.1 months for the TMZ group and 5.8 months for the nitrosourea group. Other studies have shown that the benefit of TMZ rechallenge may be restricted to patients with tumors displaying MGMT promoter methylation [3,11,46,47]. It would be interesting to compare TMZ rechallenge with other regimens in prospective randomized trials in patients with a long TFR and/or with MGMT-promoter methylated GB to determine the optimal second-line treatment for these patients.

All of our data are consistent with the findings of recent studies [9,11]. They show that none of the traditionally used regimens is unequivocally better than the others for the second-line treatment of GB. In recent years, a multitude of novel drugs, such as tyrosine kinase inhibitors and immune checkpoint inhibitors, have shown promising signs of efficacy against rGB, but the reported responses were observed in a highly selected and very limited patient population [9,11].

## 5. Limitations

This study has several limitations. It was a retrospective analysis with only small numbers of patients per group for group analyses, which may, therefore, have been subject to several unavoidable biases. Immunohistochemistry for IDH1-R132H was the only technique used for assessment of the IDH status of the tumors. Sanger sequencing for IDH1/2 genes was not systematically performed for all cases. It was not possible to assess the impact of MGMT methylation status on OS1 and OS2 because of the large amounts of missing data. An analysis of MGMT methylation status is not mandatory for routine pathology reports in the FGB network because of its minimal relevance to clinical decision-making for first-line treatment. However, knowledge of MGMT methylation status might be useful for decisions concerning second-line treatment [47,48]. Another limitation was the lack of accurate KPS score determination before first surgery and at recurrence. Audureau et al. [26] showed that a decrease in KPS at progression was a strong independent predictor of poorer OS2. The lack of formal volumetric analysis to measure preoperative tumor volume and residual volume is another limitation of our study. The data were also incomplete for the toxicity of second-line treatments. Furthermore, it was not possible to determine whether patients conserved a good quality of life after the various second-line treatments, because HRQoL is not systematically assessed outside of clinical trials. The routine assessment of HRQoL would be of great interest for clinicians needing to choose between second-line treatments with similar efficacies in terms of survival. All of these limitations highlight that corrective actions need to be taken within the FGB to have more complete eCRFs.

## 6. Conclusions and Perspectives

Within the FGB, four treatment options for rGB patients were recorded: repeat radiotherapy, reoperation, systemic treatment and inclusion in a clinical trial. Systemic treatment was the option most frequently selected for rGB patients, with bevacizumab plus nitrosourea more frequently used in patients with a short or intermediate TFR and TMZ rechallenge and bevacizumab plus TMZ more frequently used in patients with a long TFR. None of the systemic treatment regimens was unequivocally better than the others for second-line therapy. Reoperation before the administration of systemic treatment did not increase survival. Survival was best for patients with a long TFR, but these patients accounted for only a small proportion of the study population (13.0%). This better survival appeared to be more strongly associated with response to first-line treatment than with response to second-line treatment, indicating that the recurring tumors were more aggressive and/or resistant than the initial tumors in these patients. There is an urgent need for molecular investigations of the reasons for which some GB patients have a long TFR after chemoradiotherapy. With the exception of MGMT methylation status, the few studies focusing on the molecular characteristics of IDH-wildtype GB from patients in the short and long survival groups failed to identify a solid long-term survivor signature at either the genomic or transcriptomic level [49]. Additional studies based on other -omics approaches are now required, in well-designed cohorts of primary GB samples, taking into account the TFR of GB patients and their survival outcomes. Furthermore, these analyses should not be limited to tumor samples, but should also include the peritumoral brain zone and blood samples, which may contain cellular and molecular components promoting GB growth and invasion [50,51,52]. It will also be important to determine why rGB from patients with a long TFR are more aggressive and/or resistant than the initial tumor. Studies comparing differences between GB at diagnosis and at recurrence have shown that rGB generally retain the genetic and epigenetic makeup of the primary tumor and, as such, probably require the use of similar treatment regimens [9]. Our results highlight the need for further studies on the molecular characteristics and treatment responses of rGB and their initial tumors in patients with different TFR before any firm conclusions can be drawn. These future studies may facilitate the identification of prognostic and/or therapeutic clinical markers. Support from biobanks, such as the FGB, will be required, to provide the large numbers of biological samples from well-characterized patient cohorts necessary for such studies.

## Figures and Tables

**Table 1:** Demographic and clinical characteristics of IDH-wildtype GB patients with a first relapse after TMZ-based chemoradiotherapy. Patients were stratified according to TFR: short TFR (≤6 months), intermediate TFR (7 to 17 months) and long TFR (≥18 months).

	All	Short TFR	Intermediate TFR	Long TFR	*p*-Value
**Number**	338 (100.0%)	210 (62.0%)	84 (25.0%)	44 (13.0%)	
**Age (years)**					0.188
Median (range)	61 (18–81)	62 (28–81)	62 (18–79)	59 (36–79)	
≤61	172 (50.9%)	102 (48.6%)	42 (50.0%)	28 (63.6%)	
>61	166 (49.1%)	108 (51.4%)	42 (50.0%)	16 (36.4%)	
**Sex**					0.563
Male	223 (66.0%)	143 (68.1%)	53 (63.1%)	27 (61.4%)	
Female	115 (34.0%)	67 (31.9%)	31 (36.9%)	17 (38.6%)	
**Preoperative KPS (%)**					0.599
≤70	43 (12.7%)	29 (13.8%)	10 (11.9%)	4 (9.1%)	
>70	223 (66.0%)	151 (71.9%)	41 (48.8%)	31 (70.5%)	
Unknown	72 (21.3%)	30 (14.3%)	33 (39.3%)	9 (20.5%)	
**Tumor laterality**					0.009 *
Right	187 (55.3%)	112 (53.3%)	57 (67.9%)	18 (40.9%)	
Left	151 (44.7%)	98 (46.7%)	27 (32.1%)	26 (59.1%)	
**Extent of tumor**					0.002 *
Unilobar	211 (62.4%)	118 (56.2%)	56 (66.7%)	37 (84.1%)	
Multilobar	127 (37.6%)	92 (43.8%)	28 (33.3%)	7 (15.9%)	
**EOR1**					0.027 *
PR/STR	160 (47.3%)	110 (52.4%)	36 (42.9%)	14 (31.8%)	
GTR	172 (50.9%)	97 (46.2%)	45 (53.6%)	30 (68.2%)	
Unknown	6 (1.8%)	3 (1.4%)	3 (3.6%)	0 (0.0%)	
**MGMT methylation status**					<0.001 *
Without methylation	96 (28.4%)	67 (31.9%)	21 (25.0%)	8 (18.2%)	
With methylation	72 (21.3%)	31 (14.8%)	16 (19.0%)	25 (56.8%)	
Unknown	170 (50.3%)	112 (53.3%)	47 (56.0%)	11 (25.0%)	
**Adjuvant TMZ**					<0.001 *
<6 cycles	206 (60.9%)	189 (90.0%)	15 (17.9%)	2 (4.5%)	
=6 cycles	71 (21.0%)	19 (9.0%)	35 (41.7%)	17 (38.6%)	
>6 cycles	48 (14.2%)	2 (1.0%)	30 (35.7%)	16 (36.4%)	
>12 cycles	13 (3.8%)	0 (0.0%)	4 (4.8%)	9 (20.5%)	
Min-Max	0–25	0–7	0–17	1–25	
**Recurrence location**					0.021 *
Local	299 (88.5%)	186 (88.6%)	75 (89.3%)	38 (86.4%)	
Distant	21 (6.2%)	7 (3.3%)	8 (9.5%)	6 (13.6%)	
Unknown	18 (5.3%)	17 (8.1%)	1 (1.2%)	0 (0.0%)	
**Second-line options**					0.357
Supportive care	37 (10.9%)	30 (14.3%)	5 (6.0%)	2 (4.5%)	
Treatment	301 (89.1%)	180 (85.7%)	79 (94.0%)	42 (95.5%)	
*Repeat radiotherapy*	12 (3.6%)	3 (1.4%)	5 (6.0%)	4 (9.1%)	
Alone	3 (0.9%)	1 (0.5%)	1 (1.2%)	1 (2.3%)	
With reoperation +/− systemic treatment	4 (1.2%)	0 (0.0%)	2 (2.4%)	2 (4.5%)	
With systemic treatment	5 (1.5%)	2 (1.0%)	2 (2.4%)	1 (2.3%)	
*Reoperation*	65 (19.2%)	34 (16.2%)	23 (27.4%)	8 (18.2%)	
Alone	4 (1.2%)	0 (0.0%)	2 (2.4%)	2 (4.5%)	
With intratumoral treatment +/− systemic treatment	26 (7.7%)	14 (6.7%)	8 (9.5%)	4 (9.1%)	
With systemic treatment	35 (10.4%)	20 (9.5%)	13 (15.5%)	2 (4.5%)	
*Systemic treatment alone*	206 (60.9%)	135 (64.3%)	43 (51.2%)	28 (63.6%)	
*Inclusion in a clinical trial*	18 (5.3%)	8 (3.8%)	8 (9.5%)	2 (4.5%)	
**Systemic treatment regimen**					0.001 *
TMZ rechallenge	40 (11.8%)	18 (8.6%)	9 (10.7%)	13 (29.5%)	
Nitrosourea	33 (9.8%)	22 (10.5%)	7 (8.3%)	4 (9.1%)	
Bevacizumab	38 (11.2%)	26 (12.4%)	8 (9.5%)	4 (9.1%)	
Bevacizumab + TMZ	15 (4.4%)	7 (3.3%)	2 (2.4%)	6 (13.6%)	
Bevacizumab + nitrosourea	82 (24.3%)	59 (28.1%)	19 (22.6%)	4 (9.1%)	
Bevacizumab + irinotecan	35 (10.4%)	24 (11.4%)	8 (9.5%)	3 (6.8%)	
Bevacizumab + other	6 (1.8%)	5 (2.4%)	1 (1.2%)	0 (0%)	
PCV	2 (0.6%)	1 (0.5%)	1 (1.2%)	0 (0.0%)	
Carboplatin +/− etoposide	9 (2.7%)	3 (1.4%)	5 (6.0%)	1 (2.3%)	
Inclusion in a clinical trial	15 (4.4%)	7 (3.3%)	7 (8.3%)	1 (2.3%)	
**Survival outcome**					
*After first surgery*					
PFS1					<0.001 *
Median (months) (95% CI)	7.8 (6.8–8.6)	5.9 (5.4–6.3)	13.6 (12.2–14.9)	30.2 (27.4–37.1)	
PFS1–12 rate (%) (95% CI)	28.4 (24.0–33.6)	0.0 (0.0–0.0)	6.2 (5.2–7.3)	100.0 (100.0–100.0)	
OS1					<0.001 *
Median (months) (95% CI)	19.8 (18.5–22.0)	15.2 (14.4–17.1)	22.9 (21.2–25.1)	44.3 (41.7–56.4)	
OS1–36 rate (%) (95% CI)	18.2 (14.0–23.5)	5.6 (2.7–11.7)	6.8 (2.8–16.6)	78.9 (67.5–92.2)	
*After first progression*					
PFS2					<0.001 *
Median (months) (95% CI)	5.5 (4.8–6.0)	5.9 (5.1–6.9)	4.0 (3.5–4.9)	7.0 (5.5–9.1)	
PFS2–12 rate (%) (95% CI)	13.0 (9.7–17.5)	15.5 (11.0–21.8)	3.8 (1.3–11.5)	19.6 (10.6–36.4)	
OS2					0.011 *
Median (months) (95% CI)	9.9 (8.8–10.8)	9.5 (8.3–11.2)	8.5 (6.7–9.3)	13.0 (11.2–17.7)	
OS2–18 rate (%) (95% CI)	21.4 (17.0–26.9)	22.0 (16.4–29.4)	16.3 (9.6–27.6)	29.6 (18.4–47.5)	

Abbreviations: EOR1, extent of first resection, GTR, gross total resection (100%); KPS, Karnofsky performance score; MGMT, O(6)-methylguanine methyltransferase; OS1, overall survival after first surgery; OS1–36, survival rate 36 months after first surgery; OS2, overall survival after first progression; OS2–18, survival rate 18 months after first progression; PCV, procarbazine, 1-(2-chloroethyl)-3-cyclohexyl-1-nitrosourea (CCNU, lomustine) and vincristine; PFS1, progression-free survival after first surgery; PFS1–12, progression-free survival 12 months after first surgery; PFS2, progression-free survival after first progression; PFS2–12, progression-free survival 12 months after first progression; PR, partial resection (<90%); STR, subtotal resection (≥90%); TFR, time to first recurrence; TMZ, temozolomide. * *p* < 0.05.

**Table 2:** Multivariate Cox regression analysis of factors associated with OS1 and OS2 in IDH-wildtype GB patients treated with the Stupp’s regimen in the first line of treatment and subsequently receiving supportive care or treatment after progression. The Cox model for OS1 was stratified for TFR and PFS2 and the Cox model for OS2 was stratified for PFS2, to satisfy the assumption of proportional hazards.

Variable	OS1			OS2		
	OR	95% CI	*p*-Value	OR	95% CI	*p*-Value
Age (>61 years)	1.42	(1.10–1.84)	0.007 *	1.28	(0.96–1.70)	0.094
Sex (female)	0.81	(0.62–1.07)	0.136	0.77	(0.56–1.05)	0.097
KPS (>70%)				0.87	(0.57–1.34)	0.527
Tumor laterality (left)	0.96	(0.74–1.24)	0.746	0.83	(0.62–1.11)	0.205
Tumor extent (multilobar)	1.12	(0.86–1.47)	0.407			
EOR1 (GTR)	0.98	(0.76–1.27)	0.879			
TFR						
*Long*				1		
*Short*				1.44	(0.95–2.20)	0.089
*Intermediate*				1.41	(0.86–2.31)	0.172
Recurrence location (distant)				1.50	(0.86–2.62)	0.153
Second-line treatment (treatment)	0.28	(0.19–0.42)	<0.001 *	0.18	(0.11–0.29)	<0.001 *

Abbreviations: CI, confidence interval; EOR1, extent of the first resection; GTR, gross total resection (100%); KPS, Karnofsky performance score; OR, odds ratio; OS1, overall survival after first surgery; OS2, overall survival after first progression; PFS2, progression-free survival after first progression; TFR, time to first recurrence; TMZ, temozolomide. * *p* < 0.05

**Table 3:** Survival outcomes of IDH-wildtype GB patients as a function of second-line option.

Survival Outcome							
PFS1	PFS1–12	OS1	OS1–36	PFS2	PFS2–12	OS2	OS2–18
Median (months)	Rate (%)	Median (months)	Rate (%)	Median (months)	Rate (%)	Median (months)	Rate (%)
(95% CI)	(95% CI)	(95% CI)	(95% CI)	(95% CI)	(95% CI)	(95% CI)	(95% CI)
**Supportive Care (*n* = 37)**							
6.6	16.2	11.0	3.3	NA	NA	2.9	3.5
(5.2–8.6)	(7.8–33.7)	(9.4–13.2)	(0.5–22.5)	NA	NA	(2.2–4.1)	(0.5–23.5)
**Treatment (*n* = 301)**							
8.0	29.9	21.2	19.9	5.7	13.7	10.6	23.5
(7.0–8.8)	(25.2–35.6)	(19.4–23.3)	(15.4–25.8)	(5.2–6.2)	(10.2–18.6)	(9.5–11.9)	(18.7–29.5)
**Systemic Treatment Alone (*n* = 195)**							
6.7	28.2	20.5	21.9	6.0	12.8	10.8	20.1
(6.5–7.9)	(22.6–35.3)	(18.5–23.0)	(16.1–29.8)	(5.4–6.9)	(8.6–19.0)	(9.4–12.0)	(14.6–27.7)
**Reoperation + Systemic Treatment (*n* = 32)**							
8.0	28.1	21.9	15.5	5.4	13.4	10.4	27.8
(6.8–10.2)	(16.2–48.9)	(18.0–29.8)	(6.3–37.9)	(4.4–7.2)	(5.4–33.3)	(8.8–18.0)	(15.0–51.4)
**Reoperation + Gliadel^®^ (*n* = 26)**							
8.6	26.9	24.4	17.8	5.9	20.0	14.8	39.4
(5.9–12.2)	(14.3–50.7)	(18.3–33.0)	(7.3–43.2)	(4.5–8.7)	(9.1–43.8)	(8.1–21.4)	(23.7–65.5)
**TMZ (*n* = 32)**							
13.4	56.3	30.3	43.4	4.8	10.2	13.5	32.4
(6.2–22.6)	(41.4–76.4)	(23.5–44.3)	(28.3–66.5)	(3.7–8.3)	(3.5–29.7)	(12.0–20.8)	(18.7–56.2)
**Nitrosourea (*n* = 32)**							
7.6	15.6	22.2	20.5	4.6	11.1	10.6	23.2
(6.5–9.2)	(7.0–35.0)	(16.2–33.5)	(8.7–48.1)	(3.6–7.1)	(3.8–31.8)	(8.2–14.2)	(10.9–49.5)
**Bevacizumab (*n* = 36)**							
6.7	25.0	21.4	20.3	6.2	16.2	9.9	28.8
(5.5–10.4)	(14.2–44.0)	(17.6–35.4)	(9.0–45.7)	(4.6–10.8)	(6.8–38.9)	(7.5–18.7)	(15.9–52.1)
**Bevacizumab + TMZ (*n* = 14)**							
10.5	42.9	34.6	42.3	7.9	21.4	12.4	17.5
(4.5–49.8)	(23.4–78.5)	(17.3-NA)	(21.8–82.0)	(7.0–16.5)	(7.9–58.4)	(10.1–NA)	(5.0–61.2)
**Bevacizumab + Nitrosourea (*n* = 80)**							
6.7	22.5	18.2	7.0	5.6	9.0	8.8	13.4
(6.5–7.4)	(15.0–33.8)	(15.2–20.8)	(2.8–17.7)	(4.6–6.9)	(4.2–19.2)	(6.9–11.1)	(7.1–25.3)
**Bevacizumab + Irinotecan (*n* = 33)**							
6.7	24.2	18.5	20.4	7.5	19.2	10.8	18.7
(5.6–10.6)	(13.3–44.3)	(16.2–28.0)	(9.6–43.5)	(5.9–9.6)	(9.4–39.3)	(9.1–17.4)	(8.3–42.3)

Abbreviations: OS1, overall survival after first surgery; OS1–36, survival rate 36 months after first surgery; OS2, overall survival after first progression; OS2–18, survival rate 18 months after first progression; PFS1, progression-free survival after first surgery; PFS1–12, progression-free survival rate 12 months after first surgery; PFS2, progression-free survival after first progression; PFS2–12, progression-free survival rate 12 months after first progression; TMZ, temozolomide.

## Data Availability

The datasets generated and/or analyzed in this study are available from the corresponding author under the authorization of the delegation for clinical research and innovation (DRCI, CHU, Angers).

## Supplementary Materials

The following supporting information can be downloaded at: https://www.mdpi.com/article/10.3390/cancers14225510/s1, Figure S1: Survival outcomes (PFS1 and OS1) for patients on systemic treatment; Figure S2: Kaplan-Meier curves (PFS1 and OS1) for survival stratified for reoperation with Gliadel^®^ and reoperation with systemic treatment. Table S1: Univariate Cox regression analysis of factors associated with OS1 and OS2 in IDH-wildtype GB patients treated with Stupp’s regimen as the first-line treatment and receiving supportive care or treatment following progression; Table S2: Relationships between clinical variables, assessed by Spearman’s correlation analysis; Table S3: Characteristics of IDH-wildtype GB patients treated with Stupp’s regimen as the first-line treatment and receiving one of six types of systemic treatment with or without reoperation, following progression: TMZ rechallenge, nitrosourea monotherapy, bevacizumab alone or combined with TMZ, nitrosourea or irinotecan; Table S4: Characteristics of IDH-wildtype GB patients treated with Stupp’s regimen as the first-line treatment and undergoing one of two types of second-line treatment after progression: reoperation with Gliadel^®^ or reoperation followed by systemic treatment.

## Abbreviations

EOR1: extent of the first resection, GTR, gross total resection (100%); HRQoL, health-related quality of life; KPS, Karnofsky performance score; MGMT, O(6)-methylguanine methyltransferase; OS1, overall survival after first surgery; OS1–36, survival rate 36 months after first surgery; OS2, overall survival after first progression; OS2–18, survival rate 18 months after first progression; PCV, procarbazine, 1-(2-chloroethyl)-3-cyclohexyl-1-nitrosourea (CCNU, lomustine), and vincristine; PFS1, progression-free survival after first surgery; PFS1–12, progression-free survival rate 12 months after first surgery; PFS2, progression-free survival after first progression; PFS2–12, progression-free survival rate 12 months after first progression; PR, partial resection (<90%); STR, subtotal resection (≥90%); TFR, time to first recurrence; TMZ, temozolomide.

## Appendix A

**^‡^** Amiens (M. Boone; J.-M. Constans; C. Desenclos; Y.-E. Herpe; N. Guilain; R. Tebbakha): Service d’Oncologie médicale, CHU Amiens-Picardie, Amiens, France (M.B.); Neuro-radiologie, CHU Amiens-Picardie, Amiens, France (J.-M.C.); EA CHIMERE 7516, Université de Picardie Jules Verne, Amiens, France (J.-M.C.); Département de Neurochirurgie, CHU Amiens-Picardie, Amiens, France (C.D.); Biobanque de Picardie, Amiens, France (Y.-E.H.); Service d’Anatomie et Cytologie Pathologiques, CHU Amiens-Picardie, Amiens, France (N.G.); EA4667, LPCM, Université de Picardie Jules Verne, Amiens, France (N.G.; R.T.); Tumorothèque de Picardie, CHU Amiens-Picardie, Amiens, France (R.T.). Angers (O. Blanchet; P. Menei (FGB network national coordinator); A. Rousseau): CRB—BB-0033-00038, CHU Angers, Angers, France (O.B.); Département de Neurochirurgie, CHU Angers, Angers, France (P.M.); Université d’Angers, Inserm UMR 1307, CNRS UMR 6075, Nantes Université, CRCI2NA, Angers, France (P.M.; A.R.); Département de Pathologie, CHU Angers, Angers, France (A.R.). Bordeaux (S. Bouillot-Eimer; P. Dubus; H. Loiseau): Service de Pathologie, CHU Bordeaux, Site Pellegrin, Bordeaux, France (S.B.-E.); Centre de ressources biologiques en cancérologie et Service de biologie des tumeurs, CHU Bordeaux, Bordeaux France (P.D.); INSERM U1312, BRIC, BoRdeaux Institute in onCology, Université de Bordeaux, Bordeaux, France (P.D.; H.L.); Service de Neurochirurgie, CHU Bordeaux, Site Pellegrin, Bordeaux, France (H.L.). Brest (E. Lippert; I. Quintin-Roué; R. Seizeur): CRB Santé de Brest, CHU Brest, Brest, France (E.L.); Service d’Anatomie et Cytologie Pathologiques, CHU Brest, Brest, France (I.Q.-R.); Service de Neurochirurgie, CHU Brest, Brest, France (R.S.); LaTIM, INSERM, UMR 1101, SFR IBSAM, Brest, France (R.S.). Caen (E. Emery; M. Faisant; N. Rousseau): Département de Neurochirurgie, CHU Caen, Caen, France (E.E.); Service d’Anatomie et Cytologie Pathologiques, CHU Caen, Caen, France (M.F.); Tumorothèque de Caen Basse-Normandie, Caen, France (N.R.). Colmar (G. Ahle; R. Heller; F. Lerintiu; J. Voirin): Service de Neurologie, Hôpital Pasteur, Colmar, France (G.A.); Service de Microbiologie et Biologie Moléculaire, Hôpital Pasteur, Colmar, France (R.H.); Service d’Anatomie Pathologique, Hôpital Pasteur, Colmar, France (F.L.); Service de Neurochirurgie, Hôpital Pasteur, Colmar, France (J.V.); Dijon (M.-H. Aubriot-Lorton; W. Farah; A. Bonnin): Département de Pathologie, CHU Dijon, Dijon, France (M.-H.A.-L.); Département de Neurochirurgie, CHU Dijon, Dijon, France (W.F.); Centre de Ressources Biologiques Ferdinand Cabanne BB-0033-00044, Dijon-Bourgogne University Hospital, Dijon, France (A.B.). Grenoble (E. Gay; S. Lantuejoul; P. Mossuz): Université Grenoble Alpes, Service de Neurochirurgie CHU Grenoble Alpes, Grenoble, France (E.G.); Département de Biopathologie, Centre Léon Bérard, Unicancer Grenoble, France (S.L.); Université Grenoble-Alpes, Grenoble, France (S.L.); CRB CHU Grenoble Alpes (BB-0033-00069), Grenoble, France (P.M.). Ile de France: *AP-HP Henri Mondor* (B. Ghaleh; A. Marniche); *AP-HP Pitié Salpêtrière* (P. Cornu; J.-Y. Delattre; K. Hoang-Xuan; K. Mokhtari; M. Sanson; C. Dehais; C. Villa; S. Gaillard); *CH St Anne* (P. Niel; J. Pallud; P. Varlet); *HIA Percy* (D. Ricard; Y. Yordanova); *Hôpital Foch* (A.-L. Di Stefano; C. Horodyckid): Plateforme de Ressources Biologiques, AP-HP Hôpital Henri Mondor, Créteil, France (B.G.); Département de Neurochirurgie, AP-HP Hôpital Henri Mondor, Créteil, France (A.M.); Service de Neurochirurgie, Hôpital de la Pitié-Salpêtrière, AP-HP, Médecine Sorbonne Université, Paris, France (P.C.; S.G.); Sorbonne Université, Inserm, CNRS, UMR S 1127, Institut du Cerveau et de la Moelle épinière, ICM, Paris, France (J.-Y.D.; K.H.-X.; K.M.; M.S.; A.-L.D.S.); Service de Neurologie, Hôpital de la Pitié-Salpêtrière, AP-HP, Paris, France (J.-Y.D.; M.S.); Service de Neuropathologie, Hôpital de la Pitié-Salpêtrière, AP-HP, Paris, France (K.M.; C.V.); OncoNeuroThèque, Hôpital de la Pitié-Salpêtrière, AP-HP, Paris, France (K.H.-X.; K.M.); Sorbonne Université, Service Neurologie 2—Mazarin, GH Pitié-Salpêtrière, AP-HP, Paris, France (K.H.-X.; C.D.); INSERM U1016, CNRS UMR 8104, Institut Cochin, Université Paris Descartes, Paris, France (C.V.); Département de Biologie, Hôpital Sainte-Anne, ParLille (P. Gelé C.-A. Maurage; N. Reyns): CRB/CIC1403, Université de Lille, CHU Lille, Lille, France (P.G.); Service d’anatomie pathologique, centre de biologie pathologie, CHU de Lille, Lille, France (C.-A.M.); Département de Neurochirurgie, CHU Lille, Lille, France (N.R.); ONCO-THAI, INSERM U1189, Univ Lille, France (N.R.). Limoges (F. Caire; F. Labrousse; V. Fermeaux; J. Feuillard): Service de Neurochirurgie, CHU Limoges, Limoges, France (F.C.); Département de Pathologie, CHU Limoges, Limoges, France (F.L.; V.F.); Laboratoire d’Hématologie, CHU Dupuytren, Limoges, France (J.F.); Unité Mixte de Recherche CNRS 7276 and INSERM U1262, Limoges, France (J.F.). Lyon (F. Ducray; N. Dufay; J. Guyotat; D. Meyronet): Hospices Civils de Lyon, Groupe Hospitalier Est, Service de Neuro-Oncologie, Lyon, France (F.D.); Université Claude Bernard Lyon 1, Lyon, France (F.D.; D.M.); Département Plasticité de la cellule cancéreuse, Centre de Recherche en Cancérologie de Lyon, INSERM U1052, CNRS UMR5286, Lyon, France (F.D.; D.M.); NeuroBioTec, Groupement Hospitalier Est, Hôpital Neurologique Pierre Wertheimer, Lyon, France (N.D.); Hospices Civils de Lyon, Groupe Hospitalier Est, Service de Neurochirurgie Tumorale et Vasculaire, Lyon, France (J.G.); Hospices Civils de Lyon, Service d’Anatomopathologie, Lyon, France (D.M.). Marseille: *AP-HM* (O. Chinot; H. Dufour; T. Graillon; D. Figarella-Branger); *Clairval* (D. Figarella-Branger; P. Metellus): Aix-Marseille Univ, APHM, CNRS, INP, Inst Neurophysiopathol, CHU Timone, Service de Neuro-Oncologie, Marseille, France (O.C.); Département de Neurochirurgie, Hôpital de la Timone, Marseille, France (H.D.; T.G.); Aix-Marseille Univ, APHM, CNRS, INP, Inst Neurophysiopathol, CHU Timone, Service d’Anatomie Pathologique et de Neuropathologie, Marseille, France (D.F.-B.); Aix-Marseille Univ, CNRS, INP, Inst Neurophysiopathol, Marseille, France (P.M.); Ramsay Santé, Hôpital Privé Clairval, Département de Neurochirurgie, Marseille, France (P.M.). Montpellier (L. Bauchet; C. Gozé; V. Rigau): Département de Neurochirurgie, CHU Montpellier, Université de Montpellier, Montpellier, France (L.B.); Institut de Génomique Fonctionnelle (IGF), INSERM U1191, Univ Montpellier, Montpellier, France (L.B.; C.G.; V.R.); Département de Pathologie et d’Oncobiologie, CHU Montpellier, Montpellier, France (C.G.; V.R.); Tumorothèque, CRB, CHU Montpellier, Montpellier, France (V.R.). Nancy (G. Gauchotte; M. Blonski; F. Rech): Département de Pathologie, CHRU Nancy, Nancy, France (G.G.); Service de Neurologie, CHRU Nancy, Nancy, France (M.B.); Université de Lorraine, CHRU Nancy, Service de Neurochirurgie, Nancy, France (F.R.). Nantes (J.-S. Frénel; O. Kerdraon; D. Loussouarn; J.-F. Mosnier): Département d’Oncologie Médicale, ICO, Nantes, France (J.-S.F.); Tumorothèque, ICO, Saint Herblain, France (O.K.); Département de Pathologie, CHU de Nantes, Nantes, France (D.L.; J.-F.M.); Tumorothèque, CHU Nantes, France (J-F.M.). Nice (F. Almairac; V. Bourg; F. Burel-Vandenbos): Université Côte d’Azur, Hôpital Pasteur 2, Service de Neurochirurgie, Nice, France (F.A.); Université Côte d’Azur, Hôpital Pasteur 2, Service de Neuro-Oncologie, Nice, France (V.B.); Université Côte d’Azur, Hôpital Pasteur 1, Laboratoire central d’Anatomie et Cytologie Pathologiques, Nice, France (F.B.-V.). Rennes (D.C. Chiforeanu; P.-J. Le Reste; V. Quillien; B. Turlin): Service d’Anatomie et Cytologie Pathologiques, CHU Pontchaillou, Rennes, France (D.-C.C.); Département de Neurochirurgie, CHU de Rennes, Rennes, France (P.-J.L.R.); INSERM U1242, “Chemistry, Oncogenesis, Stress Signaling,” Université de Rennes, Rennes, France (P.-J.L.R.; V.Q.); Département de Biologie, Centre de lutte contre le cancer Eugène Marquis, Rennes, France (V.Q.); CHU Rennes, CRB Santé, Rennes, France (B.T.). Rouen (O. Langlois; F. Marguet; M. Quillard-Muraine): Département de Neurochirurgie, CHU Rouen, Rouen, France (O.L.); UNIROUEN, INSERM 1239, Mont-Saint-Aignan, France (F.M.); Département de Pathologie, UNIROUEN, INSERM U1245, CHU Rouen, Université de Normandie, Rouen, France (F.M.); UNIROUEN, INSERM 1073, CHU Rouen, Service de Biochimie Générale, INSERM CIC-CRB 1404, Rouen, France (M.Q.-M.). Tours (A.-M. Bergemer-Fouquet; O. Herault; I. Zemmoura): Service d’Anatomie et Cytologie Pathologiques, Hôpital Bretonneau, CHRU Tours, Tours, France (A.-M.B.-F.); Université de François Rabelais, UFR de Médecine, Tours, France (A.-M.B.-F.; I.Z.); CRB-Touraine, Tours, France (O.H.); Département de Neurochirurgie, CHU Tours, Tours, France (I.Z.); UMR 1253, iBrain, Université de Tours, Inserm, Tours, France (I.Z.).
