# Supplementary material for: Management of Recurrent Glioblastomas: What Can We Learn from the French Glioblastoma Biobank?
Source: Cancers (Basel). 2022 Nov 9;14(22):5510. doi: 10.3390/cancers14225510 (PMC9688811; doi:10.3390/cancers14225510)
Supplement: Supplementary file 1 [file cancers-14-05510-s001.zip › cancers-1980914-Supplementary.pdf]

Supplementary Materials: Management of Recurrent Glioblastomas: What Can We Learn from the French Glioblastoma Biobank?

Anne Clavreul, Lila Autier, Jean-Michel Lemée, Paule Augereau, Gwénaëlle Soulard, Luc Bauchet, Dominique Figarella-Branger, Philippe Menei and FGB network

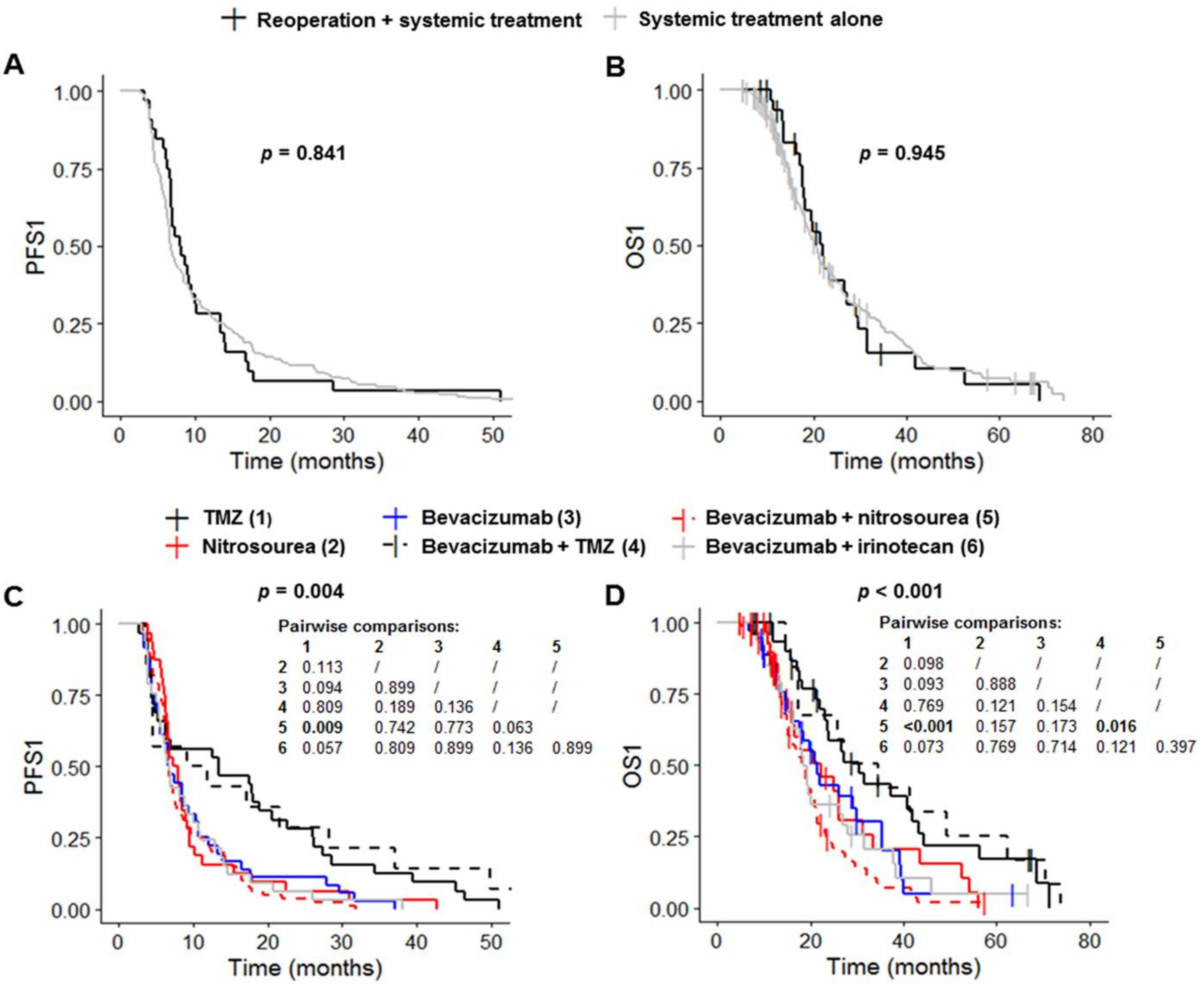

Figure S1. Survival outcomes for patients on systemic treatment. A and B) Kaplan-Meier curves for survival stratified for systemic treatment with reoperation (*n* = 32) and systemic treatment without reoperation (*n* = 195) (A: PFS1; B: OS1); C and D) Kaplan-Meier curves for survival stratified for the six most frequent systemic treatment regimens: TMZ rechallenge (*n* = 32), nitrosourea monotherapy (*n* = 32), bevacizumab alone (*n* = 36) or combined with TMZ (*n* = 14), nitrosourea (*n* = 80) or irinotecan (*n* = 33) (C: PFS1; D: OS1). Abbreviations: OS1, overall survival after first surgery; PFS1, progression-free survival after first surgery; TMZ, temozolomide.

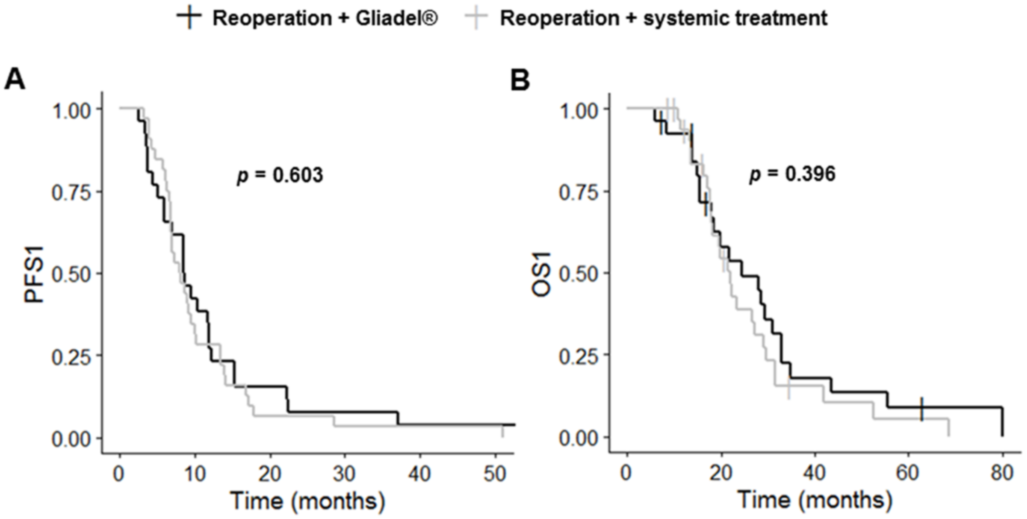

Figure S2. Kaplan-Meier curves for survival stratified for reoperation with Gliadel® (n = 26) and reoperation with systemic treatment (n = 32). A: PFS1 and B: OS1. Abbreviations: OS1, overall survival after first surgery; PFS1, progression-free survival after first surgery.

Table S1. Univariate Cox regression analysis of factors associated with OS1 and OS2 in IDH-wildtype GB patients treated with Stupp's regimen as the first-line treatment and receiving supportive care or treatment following progression.

	OS1			OS2		
**Variable**	**OR**	**95% CI**	***p*-Value**	**OR**	**95% CI**	***p*-Value**
Age (>61 years)	1.48	(1.16-1.89)	0.001 *	1.37	(1.08-1.75)	0.010 *
Sex (female)	0.84	(0.65-1.08)	0.174	0.82	(0.63-1.06)	0.123
KPS (>70%)	0.87	(0.60-1.26)	0.462	0.77	(0.53-1.10)	0.152
Tumor laterality (left)	0.84	(0.65-1.07)	0.148	0.81	(0.63-1.03)	0.084
Tumor extent (multilobar)	1.41	(1.10-1.82)	0.008 *	1.06	(0.82-1.36)	0.658
EOR1 (GTR)	0.86	(0.67-1.10)	0.220	1.04	(0.81-1.32)	0.776
MGMT status (methylated)	0.43	(0.30-0.62)	<0.001 *	0.53	(0.37-0.76)	<0.001 *
Adjuvant TMZ (≥6 cycles)	0.36	(0.28-0.46)	<0.001 *	0.90	(0.70-1.14)	0.373
PFS1 (>6 months)	0.47	(0.36-0.60)	<0.001 *	1.17	(0.91-1.50)	0.217
TFR						
Long	1					
Short	6.88	(4.61-10.26)	<0.001 *	1.55	(1.08-2.23)	0.018 *
Intermediate	3.68	(2.38-5.67)	<0.001 *	1.84	(1.23-2.75)	0.003 *
PFS2 (>6 months)	0.42	(0.32-0.55)	<0.001 *	0.27	(0.21-0.36)	<0.001 *
Recurrence location (distant)	0.95	(0.60-1.51)	0.840	1.32	(0.84-2.09)	0.231
Second-line treatment (treatment)	0.29	(0.20-0.42)	<0.001 *	0.20	(0.14-0.29)	<0.001 *

Abbreviations: CI, confidence interval; EOR1, extent of the first resection; GTR, gross total resection (100%); KPS, Karnofsky performance score; MGMT, O(6)-methylguanine methyltransferase; OR, odds ratio; OS1, overall survival after first surgery; OS2, overall survival after first progression; PFS1, progression-free survival after first surgery; PFS2, progression-free survival after first progression; TFR, time to first recurrence; TMZ, temozolomide. * $p < 0.05$.

Table S2. Relationships between clinical variables, assessed by Spearman's correlation analysis.

	**TMZ**		**MGMT**		**TFR**		**PFS1**		**PFS2**	
	**r**	***p*-Value**	**r**	***p*-Value**	**r**	***p*-Value**	**r**	***p*-Value**	**r**	***p*-Value**
**TMZ**	1.00	/	0.15	0.049 *	0.76	<0.001 *	0.61	<0.001 *	-0.03	0.541
**MGMT**	0.15	0.049 *	1.00	/	0.31	<0.001 *	0.10	0.188	0.17	0.029 *
**TFR**	0.76	<0.001 *	0.31	<0.001 *	1.00	/	0.61	<0.001 *	-0.03	0.541
**PFS1**	0.61	<0.001 *	0.10	0.188	0.61	<0.001 *	1.00	/	-0.14	0.013 *
**PFS2**	-0.03	0.541	0.17	0.029 *	-0.03	0.541	-0.14	0.013 *	1.00	/

Abbreviations: MGMT; O(6)-methylguanine methyltransferase; PFS1, progression-free survival after first surgery; PFS2, progression-free survival after first progression; TFR, time to first recurrence; TMZ, temozolomide. * $p < 0.05$.

Table S3. Characteristics of IDH-wildtype GB patients treated with Stupp's regimen as the first-line treatment and receiving one of six types of systemic treatment with or without reoperation, following progression: TMZ rechallenge, nitrosourea monotherapy, bevacizumab alone or combined with TMZ, nitrosourea or irinotecan.

	TMZ	Nitrosourea	Bevacizumab	Bevacizumab + TMZ	Bevacizumab + Nitrosourea	Bevacizumab + Irinotecan	*p*-Value
**Number**	32 (100%)	32 (100%)	36 (100%)	14 (100%)	80 (100%)	33 (100%)	
**Age (years)**							0.450
Median (range)	60 (36-79)	62 (42-77)	65 (46-76)	61 (35-79)	60 (36-78)	64 (39-79)	
≤61	20 (62.5%)	16 (50.0%)	14 (38.9%)	7 (50.0%)	42 (52.5%)	14 (42.4%)	
>61	12 (37.5%)	16 (50.0%)	22 (61.1%)	7 (50.0%)	38 (47.5%)	19 (57.6%)	
**Sex**							0.139
Male	19 (59.4%)	20 (62.5%)	19 (52.8%)	8 (57.1%)	61 (76.3%)	23 (69.7%)	
Female	13 (40.6%)	12 (37.5%)	17 (47.2%)	6 (42.9%)	19 (23.8%)	10 (30.3%)	
**Preoperative KPS (%)**							0.460
≤70	2 (6.3%)	5 (15.6%)	6 (16.7%)	1 (7.1%)	13 (16.3%)	2 (6.1%)	
>70	18 (56.3%)	20 (62.5%)	20 (55.6%)	10 (71.4%)	49 (61.3%)	27 (81.8%)	
Unknown	12 (37.5%)	7 (21.9%)	10 (27.8%)	3 (21.4%)	18 (22.5%)	4 (12.1%)	
**Tumor laterality**							0.797
Right	17 (53.1%)	17 (53.1%)	17 (47.2%)	8 (57.1%)	47 (58.8%)	21 (63.6%)	
Left	15 (46.9%)	15 (46.9%)	19 (52.8%)	6 (42.9%)	33 (41.3%)	12 (36.4%)	
**Extent of tumor**							0.366
Unilobar	18 (56.3%)	22 (68.8%)	25 (69.4%)	10 (71.4%)	43 (53.8%)	17 (51.5%)	
Multilobar	14 (43.8%)	10 (31.3%)	11 (30.6%)	4 (28.6%)	37 (46.3%)	16 (48.5%)	
**Extent of first surgery**							0.472
PR/STR	13 (40.6%)	15 (46.9%)	22 (61.1%)	7 (50.0%)	40 (50.0%)	15 (45.5%)	
GTR	19 (59.4%)	17 (53.1%)	12 (33.3%)	6 (42.9%)	38 (47.5%)	18 (54.5%)	
Unknown	0 (0.0%)	0 (0.0%)	2 (5.6%)	1 (7.1%)	2 (2.5%)	0 (0.0%)	
**Adjuvant TMZ**							0.027 *
<6 cycles	15 (46.9%)	23 (71.9%)	23 (63.9%)	6 (42.9%)	54 (67.5%)	27 (81.8%)	
≥6 cycles	17 (53.1%)	9 (28.1%)	13 (36.1%)	8 (57.1%)	26 (32.5%)	6 (18.2%)	
**MGMT methylation status**							0.184
Without methylation	4 (12.5%)	10 (31.3%)	8 (22.2%)	4 (28.6%)	33 (41.3%)	4 (12.1%)	
With methylation	11 (34.4%)	7 (21.9%)	6 (16.7%)	3 (21.4%)	17 (21.3%)	4 (12.1%)	
Unknown	17 (53.1%)	15 (46.9%)	22 (61.1%)	7 (50.0%)	30 (37.5%)	25 (75.8%)	
**TFR**							
Short	14 (43.8%)	22 (68.8%)	24 (66.7%)	7 (50.0%)	58 (72.5%)	22 (66.7%)	0.013 *
Intermediate	7 (21.9%)	7 (21.9%)	8 (22.2%)	2 (14.3%)	18 (22.5%)	8 (24.2%)	
Long	11 (34.4%)	3 (9.4%)	4 (11.1%)	5 (35.7%)	4 (5.0%)	3 (9.1%)	
**Recurrence location**							0.001 *
Local	32 (100.0%)	32 (100.0%)	31 (86.1%)	9 (64.3%)	75 (93.8%)	30 (90.9%)	
Distant	0 (0.0%)	0 (0.0%)	3 (8.3%)	4 (28.6%)	3 (3.8%)	0 (0.0%)	
Unknown	0 (0.0%)	0 (0.0%)	2 (5.6%)	1 (7.1%)	2 (2.5%)	3 (9.1%)	
**Reoperation**							<0.001 *
With	9 (28.1%)	11 (34.4%)	1 (2.8%)	1 (7.1%)	7 (8.8%)	3 (9.1%)	
Without	23 (71.9%)	21 (65.6%)	35 (97.2%)	13 (92.9%)	73 (91.3%)	30 (90.9%)	

Abbreviations: EOR1, extent of the first resection, GTR, gross total resection (100%); KPS, Karnofsky performance score; MGMT, O(6)-methylguanine methyltransferase; PR, partial resection (<90%); STR, subtotal resection (≥90%); TFR, time to first recurrence; TMZ, temozolomide. * $p < 0.05$.

Table S4. Characteristics of IDH-wildtype GB patients treated with Stupp's regimen as the first-line treatment and undergoing one of two types of second-line treatment after progression: reoperation with Gliadel® or reoperation followed by systemic treatment.

	Reoperation + Gliadel®	Reoperation + Systemic Treatment	*p*-Value
**Number**	26 (100%)	32 (100%)	
**Age (years)**			0.393
Median (range)	57 (32-72)	60 (35-74)	
≤61	19 (73.1%)	20 (62.5%)	
>61	7 (26.9%)	12 (37.5%)	
**Sex**			0.778
Male	19 (73.1%)	22 (68.8%)	
Female	7 (26.9%)	10 (31.3%)	
**Preoperative KPS (%)**			1.000
≤70	2 (7.7%)	2 (6.3%)	
>70	22 (84.6%)	22 (68.8%)	
Unknown	2 (7.7%)	8 (25.0%)	
**Tumor laterality**			0.771
Right	12 (46.2%)	16 (50.0%)	
Left	14 (53.8%)	16 (50.0%)	
**Extent of tumor**			0.405
Unilobar	19 (73.1%)	19 (59.4%)	
Multilobar	7 (26.9%)	13 (40.6%)	
**EOR1**			0.003 *
PR/STR	5 (19.2%)	19 (59.4%)	
GTR	21 (80.8%)	13 (40.6%)	
**Adjuvant TMZ**			0.635
<6 cycles	13 (50.0%)	18 (56.3%)	
≥6 cycles	13 (50.0%)	14 (43.8%)	
**MGMT methylation status**			1.000
Without methylation	9 (34.6%)	7 (21.9%)	
With methylation	4 (15.4%)	4 (12.5%)	
Unknown	13 (50.0%)	21 (65.6%)	
**TFR**			0.586
Short	14 (53.8%)	19 (59.4%)	
Intermediate	8 (30.8%)	11 (34.4%)	
Long	4 (15.4%)	2 (6.3%)	
**Recurrence location**			0.188
Local	23 (88.5%)	32 (100.0%)	
Distant	2 (7.7%)	0 (0.0%)	
Unknown	1 (3.8%)	0 (0.0%)	
**EOR2**			<0.001 *
PR/STR	3 (11.5%)	19 (59.4%)	
GTR	21 (80.8%)	13 (40.6%)	
Unknown	2 (7.7%)	0 (0.0%)	
**Systemic treatment regimen**			<0.001 *
Without	17 (65.4%)	0 (0.0%)	
TMZ rechallenge	5 (19.2%)	9 (28.1%)	
Nitrosourea	0 (0.0%)	11 (34.4%)	
Bevacizumab +/- systemic chemotherapy	4 (15.4%)	12 (37.5%)	

Abbreviations: EOR1, extent of the first resection, EOR2, extent of the second resection; GTR, gross total resection (100%); KPS, Karnofsky performance score; MGMT, O(6)-methylguanine methyltransferase; PR, partial resection (<90%); STR, subtotal resection (≥90%); TFR, time to first recurrence; TMZ, temozolomide. * $p < 0.05$.
